# Supplementary figures and images for: Mitochondrial NAD kinase Pos5 is required for CoQ biosynthesis in yeasts
Source: PLoS One. 2026 Apr 2;21(4):e0346295. doi: 10.1371/journal.pone.0346295 (PMC13046142; doi:10.1371/journal.pone.0346295)

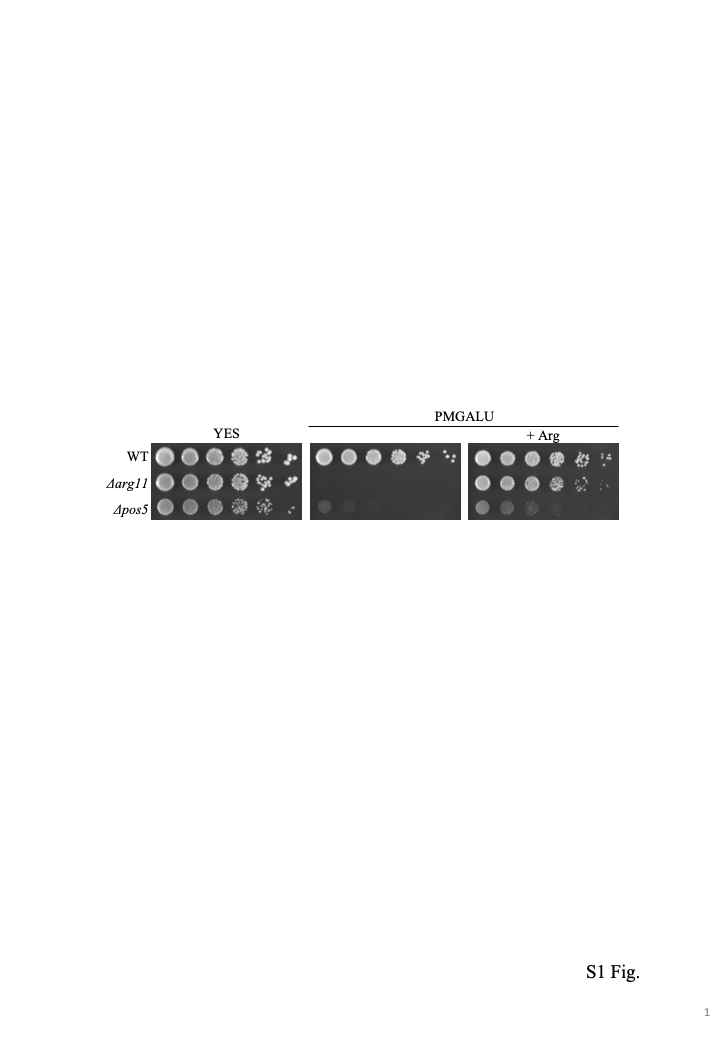

Supplement: S1 Fig — Wild-type, Δarg11, and Δpos5 strains were serially diluted (1:5) from 1 x 10^7^ cells/mL and spotted onto YES, PMGALU, and PMGALU+0.4 mg/mL arginine media. Plates were incubated at 30°C for 4 days. The Δarg11 strain, an arginine auxotroph, was included for comparison. (TIFF) [file pone.0346295.s001.tiff]

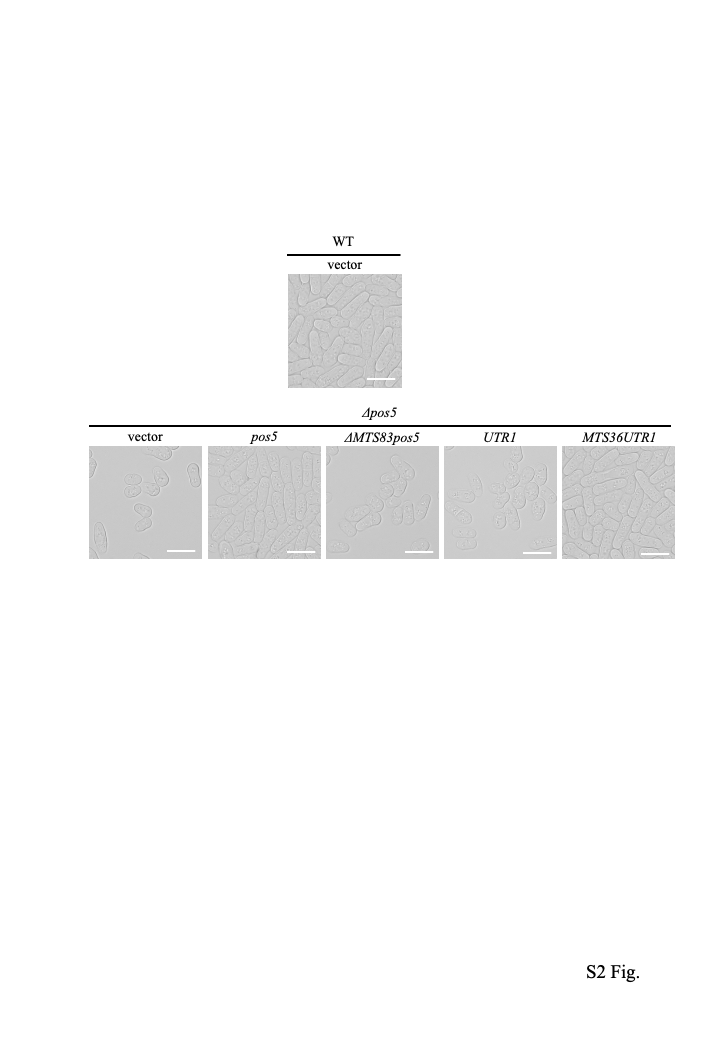

Supplement: S2 Fig — Wild-type+vector (NSP23), Δpos5 + vector (NSP26), Δpos5+pos5 (NSP11), Δpos5 + ΔMTS83pos5 (NSP15), Δpos5 + UTR1 (NSP13), and Δpos5 + MTS36UTR1 (NSP12) strains were grown at 30°C in PMLU to the mid-logarithmic phase. The cells were resuspended in PMLU and observed using a BX2-FL-2 microscope (Olympus). The scale bars indicate 10 µm. (TIFF) [file pone.0346295.s002.tiff]

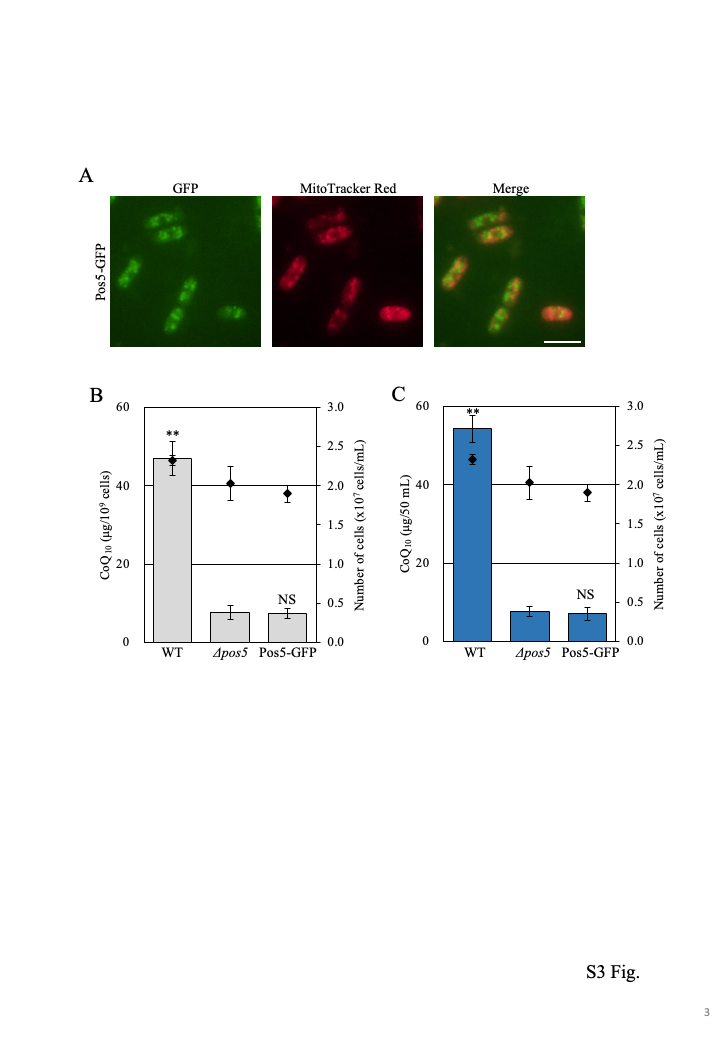

Supplement: S3 Fig — A: Localization analysis of the Pos5-GFP strain. Pos5-GFP cells were collected at mid-log phase and stained with MitoTracker Red for 1 hour. After washing, the cells were examined using fluorescence microscopy. B, C: CoQ10 quantification of the Pos5-GFP strain. Wild-type, Δpos5, and Pos5-GFP strains were cultured in YES for 48 hours. Diamonds (◆) show cell number. Bars indicate CoQ10 content per cell (B) and per volume (C). Error bars indicate the S.D. of three measurements. **: p < 0.01; statistical significance in CoQ levels (Dunnett’s test) versus the Δpos5 strain. (TIFF) [file pone.0346295.s003.tiff]

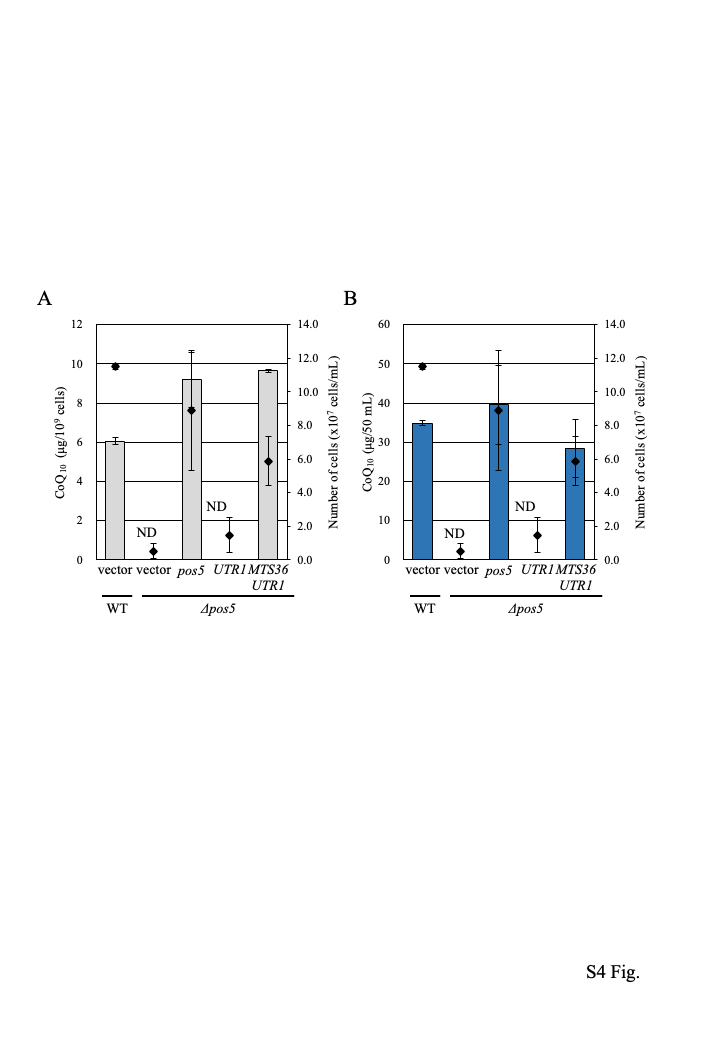

Supplement: S4 Fig — A, B: Wild-type and Δpos5 strains harboring pREP41, pREP41-pos5, pREP41-UTR1, or pREP41-MTS36UTR1 were cultured in PMU medium for 72 hours. Diamonds (◆) show cell number. Bars indicate CoQ10 content per cell (A) and per volume (B). Error bars indicate the S.D. of two measurements. (TIFF) [file pone.0346295.s004.tiff]

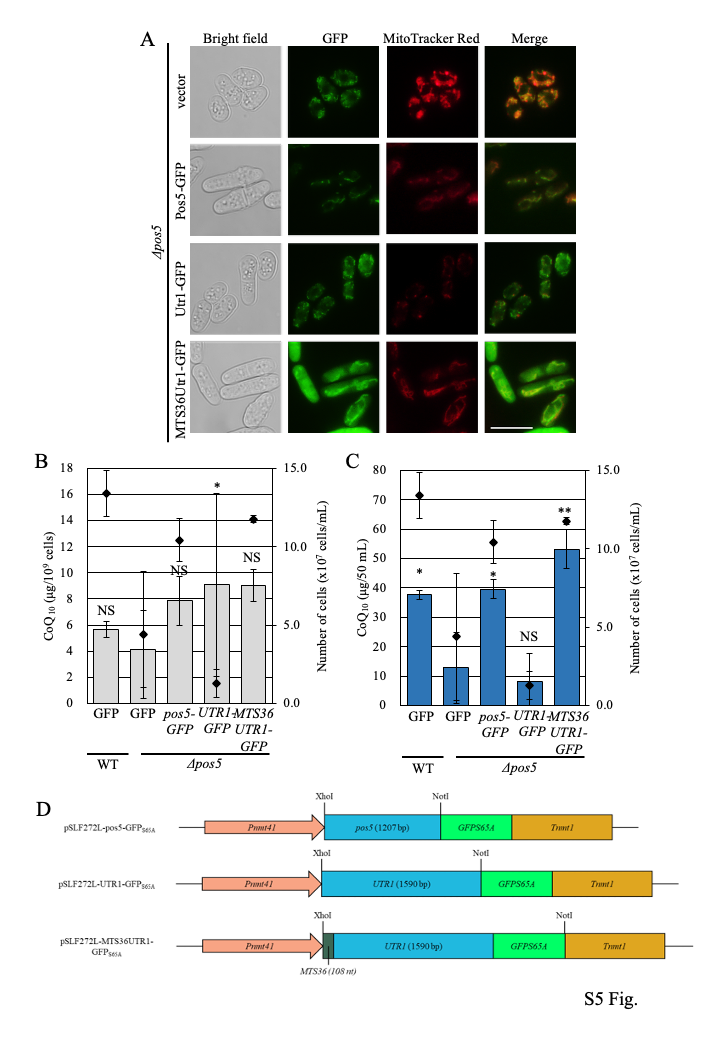

Supplement: S5 Fig — A: Fluorescent microscopy of the Δpos5 strain expressing mitochondrial or cytosolic NAD+/NADH kinase tagging with GFP at the C-terminus. Wild-type and Δpos5 strains harboring pSLF272L-GFP(S65A) or pSLF272L-UTR1-GFP(S65A) were grown at 30°C in 10 mL PMU, while Δpos5 strains harboring pSLF272L-pos5-GFP(S65A) and pSLF272L-MTS36UTR1-GFP(S65A) were grown at 30°C in 10 mL PMU + 0.1 μM thiamine. Cells were collected at 8 hours after inoculation from 5 x 10^5^ cells/mL and stained with MitoTracker Red. The scale bar indicates 10 μm. B & C: Wild-type and Δpos5 strains harboring pSLF272L-GFP(S65A), pSLF272L-pos5-GFP(S65A), pSLF272L-UTR1-GFP(S65A), or pSLF272L-MTS36UTR1-GFP(S65A) were cultured in PMU medium for 72 hours. Diamonds (◆) show cell number. Bars indicate CoQ10 content per cell (B) and per volume (C). Error bars indicate the S.D. of three measurements. *: p < 0.05; statistical significance in CoQ levels (Dunnett’s test) versus the Δpos5 strain expressing GFP. NS: no significant difference. ND: not detected. D: Plasmid map of pSLF272L-pos5-GFP(S65A), pSLF272L-UTR1-GFP(S65A), and pSLF272L-MTS36UTR1-GFP(S65A). The vector pSLF272L contains Pnmt41, GFP(S65A), and Tnmt1. (TIFF) [file pone.0346295.s005.tiff]

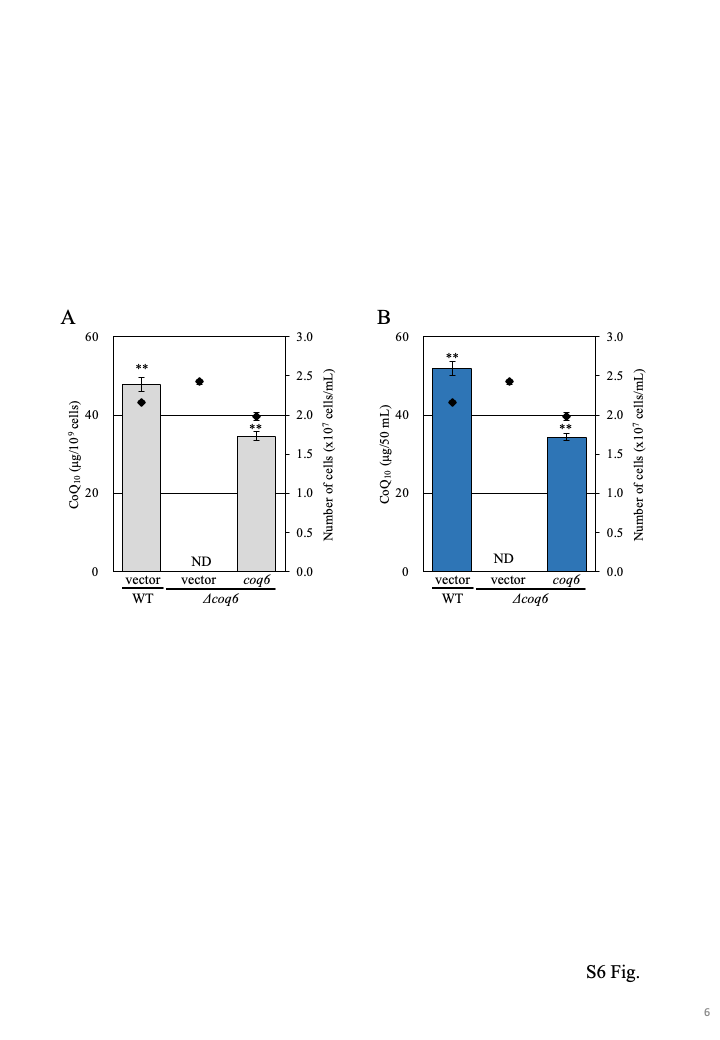

Supplement: S6 Fig — A, B: Wild-type strains integrated with the vector, Δcoq6 strains integrated with the vector, and Δcoq6 strains integrated with pJK148-Pnmt1-coq6 were cultured in YES medium for 48 hours. Diamonds (◆) show cell number. Bars indicate CoQ10 content per cell (A) and per volume (B). Error bars indicate the S.D. of three measurements. **: p < 0.01; statistical significance in CoQ levels (Dunnett’s test) versus the Δcoq6 strain integrated with the vector. ND: not detected. (TIFF) [file pone.0346295.s006.tiff]

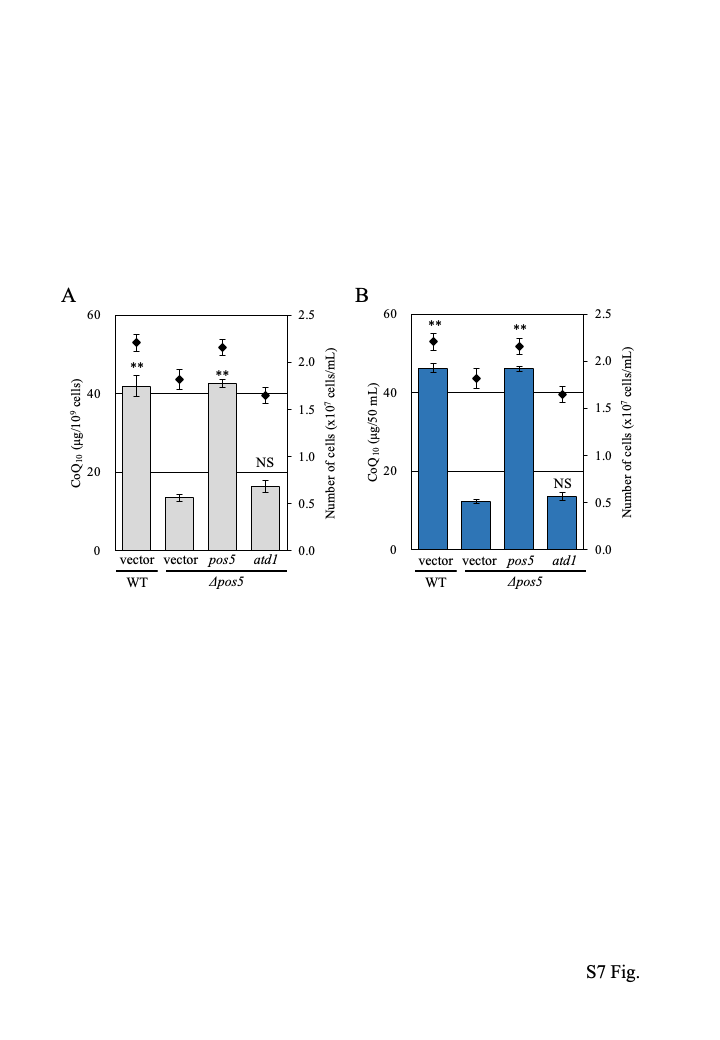

Supplement: S7 Fig — A, B: Wild-type+vector (NSP23), Δpos5 + vector (NSP26), Δpos5+pos5 (NSP11), and Δpos5 + atd1 (NSP60) strains were cultured in YES medium for 48 hours. Diamonds (◆) show cell number. Bars indicate CoQ10 content per cell (A) and per volume (B). Error bars indicate the S.D. of three measurements. **: p < 0.01; statistical significance in CoQ levels (Dunnett’s test) versus Δpos5 strain integrated with vector. NS: no significant difference. (TIFF) [file pone.0346295.s007.tiff]
