# Supplementary material for: Mitochondrial NAD kinase Pos5 is required for CoQ biosynthesis in yeasts
Source: PLoS One. 2026 Apr 2;21(4):e0346295. doi: 10.1371/journal.pone.0346295 (PMC13046142; doi:10.1371/journal.pone.0346295)
Supplement: Attachment — Submitted filename: 20260123 Answers to reviews.doc [file pone.0346295.s011.doc]

Thank you for evaluating our work and careful checking of our manuscript. We appreciate the positive responses from two reviewers. We marked the amended parts in red characters in the revised manuscript. We also extended the morphological analysis in Figure S2.

# Reviewer #1

1) The finding that the chromosomally integrated Pos5-GFP fusion did not localize correctly and was non-functional is critical but not fully explored. Was this due to the tag interfering with the mitochondrial targeting signal (MTS) or protein folding? This result should be discussed, as it serves as an important cautionary note for tagging strategies and confirms the necessity of the MTS.

Answer: The improper localization and non-functionality of the chromosomally integrated Pos5-GFP protein is a matter of our interest too. Our immunoblotting analysis detected the Pos5-GFP fusion protein in a correct size, without seeing free GFP. The fusion protein appeared to be synthesized properly rather than being degraded. Therefore, we think the C-terminal GFP tagging caused steric hindrance. The GFP tag likely interferes the protein’s folding that would prevent correct localization and proper enzymatic function. We have added this point in the result (P18, L9 and 14).

2) The manuscript uses Student's t-test for all pairwise comparisons. When multiple strains or conditions are compared to a single control (e.g., **Δpos5*+vector* in Fig 6), a one-way ANOVA with a post-hoc test (e.g., Tukey's) is more statistically appropriate. The authors should re-analyze their data using the correct statistical model or clearly justify the use of multiple t-tests.

Answer: Thank you for this suggestion. We have re-analyzed the data using a one-way ANOVA followed by Dunnett’s test for multiple comparisons against the control strain, instead of Student’s t-test. We confirmed the statistical significance using this appropriate analysis (Figures 1, 2, 4, 6, 7, and 8; Supplementary figures 3, 4, 5, 6 and 7; their legends; the method (P12, L4)).

3) The partial rescue by PHB is intriguing. The authors suggest it affects a reaction "upstream of CoQ biosynthesis." Since PHB is the direct substrate for Ppt1 (Coq2), does this imply that the Δpos5 strain has reduced PHB availability? Is the synthesis of PHB from tyrosine (or its mitochondrial import) potentially NADPH-dependent? This point warrants deeper discussion in the Results or Discussion section.

Answer: Knowledge of the PHB biosynthetic pathway in *S. pombe* is still limited. Atd1 is a candidate enzyme that converts 4-hydroxybenzaldehyde to PHB. We tested overexpression of the *atd1* gene in the *Δpos5* strain to see any effect on CoQ synthesis. The result showed slight increased CoQ levels in such a stain comparing with the one without the *atd1* expression (Fig. S7), but the difference was not statistically significant. We can not clearly conclude the involvement of NADPH in the Atd1 reaction. We have included this data and discussion in the revised manuscript (P21, L21; P25, L6).

4) The use of "Orange" and "Green" bars in the graphs, with descriptions in the figure legends, is non-standard and can be confusing. A clearer approach would be to use direct labels on the graphs or within the figure panels themselves (e.g., "CoQ10 / cell" and "CoQ10 / vol"). Additionally, the y-axes for cell number and CoQ content should be more distinctly separated or represented in a dual-axis graph with clear labeling.

Answer: Thank you for this suggestion. We revised the figures to improve readability following your suggestion. We separated the data into two distinct panels; one for CoQ content per cell and one for CoQ content per culture volume. We replaced the color-based description with grayscale and made distinct patterns to ensure the figures are easily readable (Figures 1, 2, 4, 6, 7 and 8; Supplementary figures 3, 4, 5, 6 and 7).

5) The authors correctly note that Pos5 is important but not essential, unlike core coq genes. However, they should more explicitly discuss whether the CoQ deficiency is a direct consequence of low NADPH for biosynthesis or an indirect effect of general mitochondrial dysfunction (e.g., impaired Fe-S cluster biogenesis, which is also Pos5-dependent). The data with the Δcyc1 mutant argue against a general respiratory defect, but other mitochondrial processes could be involved.

Answer: While we cannot rule out a possibility that Pos5 deficiency indirectly affects CoQ levels via general mitochondrial dysfunction (e.g., impaired Fe-S cluster biogenesis), our data argue against this being the primary cause. Specifically, if the defect was solely due to the dysfunction of Fe-S cluster-dependent enzymes (such as Coq6, which requires ferredoxin and Arh1), supplementation of the upstream precursor PHB in the ∆pos5 strain should not rescue CoQ levels. The observation that PHB restores CoQ biosynthesis implies that the core biosynthetic machinery downstream of PHB is functional, and that the primary bottleneck lies in the supply of the precursor. We added this point in the revised manuscript (P24, L15)

# Reviewer #2

1) The authors conclude that Pos5/NADPH is required for an "earlier step" in CoQ biosynthesis, based on the partial rescue by PHB and vanillic acid (VA) and the lack of effect from *coq6* overexpression. This is a reasonable hypothesis. However, to strengthen this conclusion, it would be informative to directly measure the levels of early intermediates (e.g., decaprenyl-PHB) in the *Δpos5* mutant compared to wild-type and perhaps a coq6 mutant. This could more precisely pinpoint the bottleneck.

Answer: This is an important point. We attempted to detect early intermediates, such as 3-decaprenyl-4-hydroxybenzoate (4-HP_10_), in the *Δpos5* strain using LC-MS/MS. However, the levels of these intermediates were below the limit of detection under our current experimental conditions. Further optimization of extraction and detection methods is required to profile these trace intermediates, which remains to be a subject for future investigation.

2) Figure 3 shows a decrease in total mitochondrial NADP(H) in the S. pombe Δpos5 strain. The claim that "NADP+ showed the most pronounced reduction" is made in the text (Page 29), but the graphical presentation in Fig. 3A (stacked bars) makes it difficult for the reader to independently assess the relative changes in NADP+ vs. NADPH. Presenting these as separate bar graphs or including the numerical values in a supplementary table would enhance clarity and support the statement regarding species-specific differences with S. cerevisiae.

Answer: As suggested, we have modified Figure 3 to present the total NADP(H) levels and the specific redox states (NADP^+^ and NADPH) in separate graphs to allow for easier comparison (New Fig. 3).

3) The discussion proposes Atd1 as a potential NADPH-dependent link in the quinone precursor pathway (Page 30). This is an interesting speculation. Could the authors provide any preliminary data or genetic interaction (e.g., double mutant analysis, overexpression of atd1 in Δpos5) to test this hypothesis? If not, the text should more clearly frame this as a suggested model for future investigation.

Answer: We performed the suggested experiment. Overexpression of *atd1* in the *Δpos5* strain slightly increased CoQ levels, although it was not statistical significance (*p* > 0.05). We have included these results in Supplementary figure S7 and added this result in the result (P21, L21)

4) Several figures (e.g., Figs 1, 2, 4, 6-8) use a dual Y-axis format (cell number and CoQ content). While informative, the graphs are somewhat crowded. Ensuring high resolution and clear differentiation of data series in the final version is essential.

Figure 5 (localization) lacks a scale bar in the provided image. This must be added.

The labels "Orange bars" and "Green bars" in the figure legends refer to colors not visible in the grayscale PDF. Please use patterns (hatching, shading) or direct labeling (e.g., "left axis: CoQ10 per 10^9 cells; right axis: CoQ10 per 50 mL culture") to ensure accessibility.

Answer: Thank you for this suggestion. Please refer to our response to Reviewer #1, Point 4. We separated the graphs to resolve the crowding issue.

5) The manuscript states the use of Student's t-test. For all multi-group comparisons (e.g., Fig. 6, 7, 8), please confirm that the appropriate statistical test (e.g., ANOVA with post-hoc test) was applied where applicable, and specify which groups are being compared when significance is indicated.

Answer: Please refer to our response to Reviewer #1, Point 2. We re-analyzed the data using ANOVA followed by Dunnett’s test.

Minor Points:

Thank you for careful checking of our manuscript. We corrected according to your suggestion.

Abstract: The phrase "inability to grown on non-fermentable carbon sources" should be corrected to "inability to grow".

Answer: We corrected (P2, L13).

Page 9, Abstract & Page 19, Results: "CoQ10 level in ΔScpos5 were decreased..." should be "was decreased" or "levels were decreased".

Answer: We corrected (P2, L11; P20, L17).

Page 12: "Synthetic defied (SD) medium" should be "Synthetic defined (SD) medium".

Answer: We corrected (P5, L16).

Page 15: "...primers containing restriction sites from the S. pombe PR110 genome and the S. cerevisiae BY4741 genome as the template." The phrasing is slightly ambiguous. Consider: "...using the S. pombe PR110 genome and the S. cerevisiae BY4741 genome as templates, with primers containing restriction sites."

Answer: We corrected (P8, L15).

Page 19: "CoQ levels in the Δpos5 strain of our construct were decreased to 0.2-fold of thoes in the wild-type..." should be "those".

Answer: We corrected (P17, L10).

Page 30: "...sulfide accumulation is not enhanced CoQ deficiency." Should likely be "is not enhanced by CoQ deficiency."

Answer: We corrected (P24, L11).

References: The reference list appears comprehensive. Please ensure all in-text citations have a corresponding entry and vice-versa.

Answer: We checked all citations.
