# Supplementary material for: Mitochondrial NAD kinase Pos5 is required for CoQ biosynthesis in yeasts
Source: PLoS One. 2026 Apr 2;21(4):e0346295. doi: 10.1371/journal.pone.0346295 (PMC13046142; doi:10.1371/journal.pone.0346295)
Supplement: Attachment — Submitted filename: renamed_7321a.docx [file pone.0346295.s012.docx]

The manuscript is clearly written and well illustrated. The conclusions are supported by the results. The authors seem to have addressed most reviewers’ comments. I only have a few minor comments.

Ans. Thank you for careful checking of our revised manuscript. Your comments help to improve the accuracy of the description. We answered the comments and explained the revised points.

P3 L9 : the claim that « most prokaryotes synthesize CoQ endogenously » is incorrect, as CoQ biosynthesis is restricted to bacteria belonging to the phylum Pseudomonadota (doi : 10.1101/2025.09.17.676790). All other bacterial phylla, nor archea, do not produce CoQ.

Ans. As you pointed out, we changed this part to ‘’belonging to the phylum Pseudomonadota’ and added the reference

P10 L18 : « OD600 = 1 point ». This seems strange. You can use « OD600 = 1 »

Ans. Yes, we changed so (P10 L17).

P10 L19 « Mitochondria were isolated according to a method described previously [27] with slight modification. ». The modifications should be indicated.

Ans. We added the description of the modified point in P10, L19.

Fig 2A : red is used to display conserved residues between the two sequences. Therefore, the amino acids of two insertions which are found only in the S. cerevisiae sequence should be displayed in a different color than red.

Ans. We changed according to your suggestion (New Figure2A).

Figure legends are long and repetitive (for example fig 1A-B, fig 2B-C, fig 6A-B, fig 7A-B). Many details could be placed in the methods section to shorten the figure legends.

Ans. We agreed. We delete some sentences and moved to M&M. (Fig 1A-B, Fig 2B-C, Fig 6A-B, Fig 7A-B).

P14 L3 : when first talking about UTR1, please explain the function of this protein.

Ans. We added the description (P14 L1).

P20 L14-16 : « Coq6 catalyzes C5-hydroxylation of the quinone precursor and requires reducing equivalents from NAD(P)H, through ferredoxin and ferredoxin reductase [35, 36]. » References 35 and 36 do not support this claim. The papers that show this are the following doi : 10.1016/j.chembiol.2011.07.008 and 10.1002/cbic.202300738

Ans. You are right. We added the appropriate references (P20 L4).

P20 L21 « However, CoQ levels of per cells and per volume » delete « of »

Ans. We amended this part and also the other parts (P20 L16 and else)

P21 L13 « clearly restored the CoQ a. » please correct to « clearly restored the CoQ level. »

Ans. We amended this part (P20 L23).
